# Natural antagonistic flavones for AhR inhibit indoxyl sulfate-induced inflammatory gene expression *in vitro* and renal pathological damages *in vivo*

**DOI:** 10.29219/fnr.v68.10032

**Published:** 2024-07-31

**Authors:** Tomomi Iwashima, Yui Takemura, Yoshimi Kishimoto, Chihiro Ono, Ayano Watanabe, Kaoruko Iida

**Affiliations:** 1 Department of Food and Nutritional Sciences, Graduate School of Humanities and Sciences, Ochanomizu University, Tokyo, Japan; 2 Department of Food Science and Human Nutrition, Setsunan University, Osaka, Japan;; 3 Institute of Human Life Science, Ochanomizu University, Tokyo, Japan

**Keywords:** aryl hydrocarbon receptor, chrysin, endothelium, flavone, indoxyl sulfate, inflammation, renal failure

## Abstract

**Background:**

Uremic toxin indoxyl sulfate (IS) induces vascular inflammation, a crucial event in renal failure, and vascular complications in patients with chronic kidney disease (CKD). In endothelial cells, IS increases the production of inflammatory cytokines partially via the activation of the aryl hydrocarbon receptor (AhR), and several food flavonoids have been reported to act as antagonists of AhR.

**Objective:**

This study aimed to investigate whether antagonistic flavonoids can attenuate IS-induced inflammatory responses in vascular endothelial cells *in vitro* and renal failure *in vivo*.

**Design:**

Human umbilical vein endothelial cells (HUVECs) pretreated with the flavones apigenin, chrysin, or luteolin were stimulated with IS. Expression levels of genes involved in AhR signaling, inflammatory cytokine production, and reactive oxygen species (ROS) production were analyzed. Uninephrectomized mice were orally administered chrysin and received daily intraperitoneal injections of IS for 4 weeks.

**Results:**

In HUVECs, IS upregulated the mRNA expression of AhR-targeted genes (*CYP1A1* and *AhRR*), and genes involved in inflammation (*NOX4*, *MCP-1*, *IL-6,* and *COX2*) and monocyte invasion/adhesion (*ICAM1*). All three flavones attenuated the IS-induced increase in the expression of these mRNAs. They also suppressed the IS-induced nuclear translocation of AhR and intracellular ROS production. Furthermore, IS-induced phosphorylation of the signal transducer and activator of transcription 3 (STAT3) was inhibited by treatment with these flavones. The results of *in-vivo* experiments showed that administration with chrysin attenuated the elevation of blood urea nitrogen levels and AhR-target gene expression and the pathological impairment of renal tissues in mice, regardless of higher serum levels of IS.

**Conclusions:**

Natural food flavones antagonizing AhR exerted protective effects against IS-induced inflammation through the inhibition of the AhR–STAT3 pathway in HUVECs. Moreover, chrysin ameliorated IS-induced renal dysfunction in a mouse model of CKD. These flavonoids could be a therapeutic strategy for vascular inflammation in CKD.

## Popular scientific summary

- Uremic toxin indoxyl sulfate (IS) causes vascular inflammation via the aryl hydrocarbon receptor (AhR), resulting in renal failure.
- Food flavonoids such as apigenin, chrysin, and luteolin exerted protective effects against IS-induced inflammation by inhibiting the AhR–STAT3 pathway in endothelial cells.
- Chrysin ameliorated IS-induced renal histological damage in a mouse model of chronic kidney disease (CKD).
- These flavonoids could be a therapeutic strategy for targeting vascular inflammation in CKD.

Chronic kidney disease (CKD) is an increasingly recognized global public health concern. CKD directly affects long-term morbidity and mortality through the progression of renal dysfunction and its associated cardiovascular risk. Approximately half of the patients with severe CKD die from cardiovascular disease (1). In patients with CKD, various toxic metabolites, called uremic toxins, accumulate in the blood because of the loss of renal function (2, 3). Among these uremic toxins, protein-bound uremic toxins such as indoxyl sulfate (IS), p-cresyl sulfate (PCS), and indole-3 acetic acid (IAA) have emerged as potent toxins that play critical roles in the progression of kidney dysfunction and cardiovascular diseases (3, 4). Thus, the removal of these toxins from the blood flow is very important; however, it is difficult to remove these toxins because of their high binding affinity to proteins.

Vascular endothelial cells are continuously exposed to uremic toxins. In the last two decades, several clinical studies have demonstrated the association between deleterious effects of protein-bound uremic toxins and vascular complications in patients with CKD (5–8). Many *in-vitro* and *in-vivo* studies have demonstrated the direct effects of uremic toxins or serum from patients with CKD on vascular cells (7, 9, 10). Moreover, the effects of IS and PCS on vascular cells have been extensively examined. IS and PCS increase the expression of proinflammatory cytokines and chemokines, such as NADPH oxidase 4 (NOX4), monocyte chemoattractant protein-1 (MCP-1), interleukin-6 (IL-6), and cyclooxygenase-2, as well as reactive oxygen species (ROS) production in vascular endothelial and smooth muscle cells (11–16). These toxins alter the expression of molecules involved in cell adhesion, such as intercellular adhesion molecule-1 (ICAM-1), vascular cell adhesion molecule-1 (VCAM-1), and E-selectin, which promote the infiltration of activated macrophages into the endothelium (7, 12, 17, 18). Endothelial dysfunction caused by immune cells or inflammatory cytokines leads to subsequent kidney failure because the kidney contains many types of endothelium, including glomerular endothelium, peritubular capillary endothelium, and endothelium of blood vessels that feed renal tissues (19).

Several mechanisms have been postulated through which uremic toxins adversely affect the vascular endothelium, including the aryl hydrocarbon receptor (AhR) pathway. AhR is a nuclear receptor that was initially discovered to mediate the toxic response to dioxin such as 2,3,7,8-tetrachlorodibenzo-p-dioxin (TCDD), which has carcinogenic and teratogenic effects (20–23). Evidence accumulated in recent years has shown that indolic uremic solutes, such as IS and IAA, bind to and activate AhR not only *in vitro* (24–26) but also *in vivo* (27–29), leading to activation of the inflammatory signaling pathway. Indeed, several experimental studies have demonstrated that IS and IAA induce the expression of pro-inflammatory genes by activating AhR/NF-κB cascades in endothelial cells (13, 30, 31), vascular smooth muscle cells, and macrophages (13, 32, 33).

To protect against the toxic effects induced by ligand-binding AhR activation, numerous investigations have been conducted to identify AhR antagonists. Several studies have indicated that polyphenolic compounds present in natural food products inhibit AhR activation. Amakura et al. tested 39 food extracts using an *in-vitro* bioassay and found that some of them competitively inhibited TCDD-induced activation of AhR (34). Moreover, certain flavones and flavonols present in food and medicinal plants have been reported to be potent candidates that antagonize the TCDD-induced activation of AhR (35, 36). Nevertheless, few studies have investigated the inhibitory effects of food-derived polyphenolic compounds on uremic toxin-induced AhR activation.

In the present study, we aimed to explore the effects of these flavones on IS-induced inflammation in vascular endothelial cells involved in the progression of chronic renal failure. We selected three flavones (apigenin, chrysin, and luteolin) with potent inhibitory effects on TCDD-induced AhR activation (36). Additionally, we conducted *in-vivo* experiments to examine whether the administration of chrysin, a flavone used in *in-vitro* study, ameliorates chronic renal injury induced by IS in mice subjected to uninephrectomy as a model of CKD.

## Materials and methods

### Materials

IS and chrysin were purchased from Cayman Chemical (Ann Arbor, MI, USA). RPMI-1640 medium, Hank’s balanced salt solution (HBSS), and 3-(4,5-dimethylthiazol-2-y1)-2,5-diphenyltetrazoli-umbromide (MTT) were purchased from Sigma-Aldrich (St. Louis, MO, USA). Fetal bovine serum (FBS), penicillin/streptomycin, novoheparin, and recombinant human fibroblast growth factor (hFGF) basic were obtained from Biowest (Nuaillé, France), GIBCO (Grand Island, NY, USA), Mochida Pharmaceutical (Tokyo), and R&D Systems (Minneapolis, MN, USA), respectively. The polyphenolic compounds apigenin, chrysin, and luteolin were purchased from Sigma-Aldrich; 5-(And-6)-chloromethyl-2ʹ,7ʹ-dichlorohydrofluorescein diacetate (CM-H2DCFDA) was obtained from Thermo Fisher Scientific (Waltham, MA, USA).

### Cell culture and treatment

Human umbilical vein endothelial cells (HUVECs) were obtained from Lonza (Basel, Switzerland) and grown in the RPMI-1640 medium supplemented with 10% FBS, 1% penicillin/streptomycin, 0.5% novoheparin, and 0.05% recombinant hFGF basic at 37°C and 5% CO_2_. HUVECs were seeded on 6-well plates (Corning, Tokyo, Japan) at a density of 2.0 × 10^5^ cells/well and on 24-well plates (Corning) at a density of 5.0 × 10^4^ cells/well for gene expression and ROS measurements, respectively. Two days later, the medium was replaced with fresh medium containing one of the three flavones – apigenin, chrysin, or luteolin (10 μM) – or equal volumes of dimethyl sulfoxide (DMSO; FUJIFILM Wako Pure Chemical, Osaka, Japan) as a vehicle control; the cells were then pretreated for 30 min. The medium was removed again, and the cells were further incubated using medium with IS (0.5–1 mM) for 6 h.

In the MTT assay, which measures cell viability, HUVECs were seeded on 24-well plates (Corning) at a density of 5.0 × 10^4^ cells/well and incubated for 48 h. The cells were then treated with medium containing IS (1 mM) or IS with one of the three flavones (10 μM) for 6 h. Later, the medium was replaced with 0.5 ml of fresh medium containing MTT (0.5 mg/ml) and incubated for an additional 3 h. The culture medium was then carefully removed, and the resulting formazan crystals were dissolved in DMSO (250 μl/well). Absorbance was measured at 570 nm using a microplate reader (BioTek Instruments, Tokyo, Japan).

### Real-time RT-PCR analysis

Total RNA was extracted from cells or tissues using the RNAiso Plus total RNA extraction reagent according to the manufacturer’s protocol (Takara Bio, Shiga, Japan). We synthesized complementary DNAs (cDNAs) from 2 μg of total RNA using a High Capacity cDNA Reverse Transcription Kit (Thermo Fisher Scientific). Real-time polymerase chain reaction (PCR) was performed using Power SYBR Green PCR master mix (Thermo Fisher Scientific) on 25 ng of synthesized cDNA with 10 μL of reaction solution under the following conditions: 95°C for 15 s, followed by 60°C for 45 s and cycling for 40 times on a StepOnePlus system (Applied Biosystems, Foster City, CA, USA). Gene products were expressed in terms of mRNA levels and normalized to a standard housekeeping gene (*r18S* RNA for *in-vitro* experiments or *Gapdh* for *in-vitro* experiments) using the ∆∆CT method. Experiments were conducted in duplicate and repeated at least twice independently. Primer sequences are listed in Supplemental Table S1.

### Western blot analysis

Total cell protein, and cytosolic and nuclear protein, were extracted using the M-PER Mammalian Protein Extraction Reagent (Thermo Fisher Scientific) and NE-PER Nuclear and Cytoplasmic Extraction Reagents (Thermo Fisher Scientific) respectively, according to the manufacturer’s protocol. Equal amounts of cellular proteins (10 μg per lane) were separated by 10% sodium dodecyl sulfate-polyacrylamide gel electrophoresis (SDS-PAGE) at 100 V for 1 h, and the protein bands were transferred to an Immobilon-P membrane (Millipore, Bedford, MA, USA) at 160 mA for 1 h. The membranes were blocked with Blocking One-P (Nakalai tesque, Kyoto, Japan) for 20 min at room temperature and incubated overnight at 4°C with the following primary antibodies at a 1:1000 dilution in Blocking One-P solution: anti-AhR, anti-laminB (Santa Cruz Biotechnology, Santa Cruz, CA, USA), anti-Stat3, anti-phospho-Stat3, anti-extracellular signal regulated kinase (ERK), anti-phospho-ERK, anti-p38, anti-phospho-p38, and anti-β-actin (Cell Signaling Technology, Danvers, MA, USA). Each bound antibody was detected by incubation for 1 h at room temperature with horseradish peroxidase-conjugated anti-rabbit or anti-mouse IgG secondary antibody (1:2000; Cell Signaling Technology) in Tris-buffered saline containing 0.1% Tween 20. Chemiluminescent detection of specific proteins was conducted using ECL Select Western Blotting Detection Reagent (GE Healthcare, Little Chalfont, UK). All signals were detected using an ImageQuant LAS-4000 luminescence image analyzer (GE Healthcare). The optical density of each band was analyzed using ImageJ (National Institutes of Health, Bethesda, MD, USA). Experiments were repeated at least twice independently.

### Measurement of Intracellular ROS Production

Intracellular ROS levels were quantified by measuring the oxidative conversion of cell-permeable CM-H2DCFDA to the fluorescent product dichlorofluorescein (DCF), following a method reported previously (37), with modifications. Fifty micrograms of CM-H2DCFDA was dissolved in HBSS to a final concentration of 10 μM as the working solution. After washing the cells with HBSS, 500 μL of the DCFH-DA working solution was added and incubated for a further 30 min at 37°C. The working solution was removed, the cells were washed once with HBSS, and fresh HBSS was added to each well. Fluorescence images were acquired using an all-in-one fluorescence microscope (BZ-X710, 20 × objective lens, KEYENCE, Osaka, Japan) equipped with a GFP filter (ex:470/40 nm, em:525/50 nm). Three images were randomly captured for each dish (*n* = 3, a total of nine images in each group). Fluorescence intensities of positive areas in each image were automatically calculated using a hybrid cell count application (BZ-H3C, KEYENCE) and the analyzer software (BZ-X Analyzer, KEYENCE) associated with the microscope.

### Animal experiment

C57BL/6 male mice (12 weeks old, *n* = 22; Japan SLC, Hamamatsu, Japan) were randomly assigned to three groups: ‘control (CON) group’ (*n* = 7); ‘IS group’ (*n* = 7); and ‘IS + C group’ (*n* = 8). Mice in the IS and IS + C groups were subjected to left total nephrectomy, while mice in the CON group were subjected to a sham operation. From the first week after surgery, IS (100 mg/kg body weight [BW]) dissolved in 0.9% saline was administered every weekday by intraperitoneal injection to mice in both the IS and IS + C groups for 4 weeks. An equivalent volume of saline was administered to mice in the CON group. Furthermore, chrysin (50 mg/kg BW) dissolved in 1% DMSO was administered by oral gavage to mice in the IS + C group 30 min before the intraperitoneal injection. Mice in the other two groups were treated with 1% DMSO as a vehicle control. After 5 weeks, the mice were anesthetized with isoflurane to collect blood samples; they were then sacrificed, and the right kidney was harvested. All animal procedures were approved by the Animal Ethics Committee of the Ochanomizu University.

### Serum biochemistry

Serum samples were isolated from the collected blood by centrifugation at 8,000 rpm for 15 min at 4°C. The blood urea nitrogen (BUN) concentration was measured using the urease and glutamate dehydrogenase (GLDH) method with Wako L-type UN reagent (Wako Pure Chemicals, Osaka, Japan). The serum creatinine concentration was determined using the LabAssay Creatinine kit (Wako Pure Chemicals). Serum IS concentrations were determined using high-performance liquid chromatography (HPLC). The HPLC apparatus included a pump (LC-20AD, Shimadzu, Kyoto, Japan), a fluorescence detector (RF-20A, Shimadzu), and an autosampler (SIL-20AC, Shimadzu). The Shim-pack XR-Phenyl (3.0 × 75 mm, 2.2 μm; Shimadzu) column was equipped with a guard column (5 μm, 4.0 × 10 mm; OSAKA SODA, Osaka, Japan). A degassed and filtered mixture of potassium dihydrogen phosphate (0.1 M) and tetrahydrofuran (95: 5, v/v) with a pH of 6.5 was used as the mobile phase. The flow rate was 0.5 ml/min at a column temperature of 40 °C, and the detector settings were Ex 295 nm/Em 390 nm.

### Histopathological analysis

Kidney tissues from mice were fixed with 3.7% formaldehyde for more than 24 h at room temperature, embedded in paraffin, and cut into 4-μm sections. The sections were stained with Periodic Acid–Schiff (PAS) or Azan. Briefly, following deparaffinization, the sections were incubated with 0.5% periodic acid solution for 10 min, and then stained with Schiff’s reagent for 30 min in PAS staining. In Azan staining, the deparaffinized sections were stained with azocarmine G for 120 min, treated with 5% phosphotungstic acid overnight, and then stained with Mallory’s anilinblue-orange-G mixture for 30 min. The stained sections were viewed under a BZ-X710 fluorescence microscope (40 × objective lens). Five fields were examined on each kidney slide. The degree of morphological damage was determined in the PAS-stained sections. The extent of morphological damage was scored using a semi-quantitative scale of 1 to 4 (1 = normal, 2 = mild, 3 = moderate, and 4 = severe) by two investigators in a double-blinded manner. The following parameters were considered indicative of morphological damage: brush border loss, tubule dilatation, tubule degeneration, tubule necrosis, cast formation, and dilation of the Bowman’s capsule. Fibrotic areas (stained blue) were quantified in the Azan-stained sections using the analysis software (BZ-X Analyzer; Keyence) associated with the microscope.

### Statistical analysis

Values are presented as mean ± standard error (SE). We performed one-way analysis of variance (ANOVA) followed by Tukey’s post-hoc test to compare the treatment groups. Statistical significance was set at *P* < 0.05. All results were analyzed using the GraphPad Prism 5 software package (GraphPad Software, La Jolla, CA, USA) and SPSS Statistics version 24 for Windows software (SPSS Inc., Chicago, IL, USA) for cell and animal experiments, respectively.

## Results

### Treatment with IS and the selected flavones did not affect cell viability in HUVECs

**Fig. 1:**
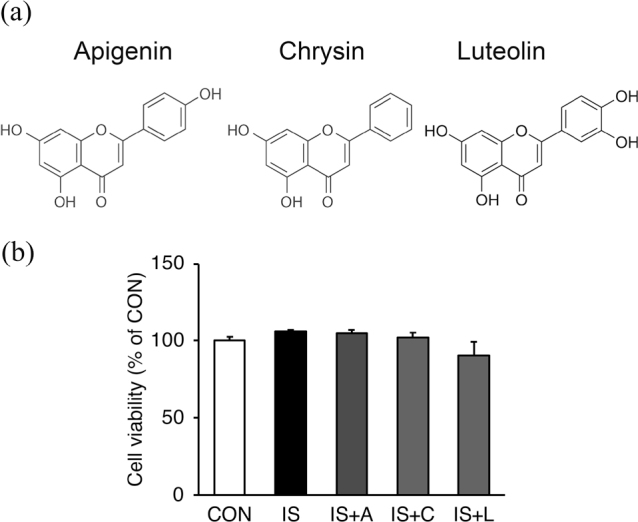
Cytotoxic effect of IS only or IS with each flavone on HUVECs. (a) Structure of the flavones used in the study. (b) The cells were treated with 1 mM IS (IS) or IS with 10 μM apigenin, A; chrysin, C; or luteolin, L; for 6 h. Cell viability was then determined by MTT assays. Values are expressed as the percentage of those of the vehicle control group (untreated cells [CON]) that was arbitrarily set to 100%. Data are the mean ± SE (*n* = 4).

The structure of flavones used in this study is shown in Fig. 1a. We first analyzed the effects of IS and the indicated flavones on the viability of HUVECs using the MTT assay. As shown in Fig. 1b, no cytotoxic effects were observed when the cells were exposed to IS only (1 mM) or IS and each of the three flavones, apigenin, chrysin, or luteolin (10 μM) for 6 h.

### Apigenin, chrysin, and luteolin reduced the expression of AHR target genes and nuclear translocation of AHR in IS-stimulated HUVECs

**Fig. 2:**
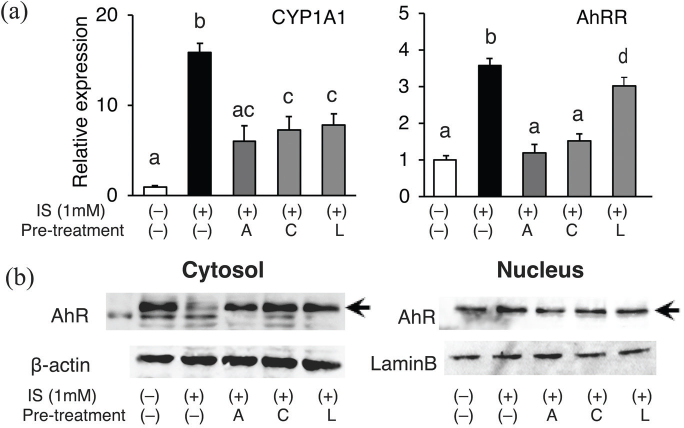
Effects of IS and each flavone on AhR-targeted gene expression. HUVECs were pretreated with the indicated flavones (apigenin; A, chrysin; C, luteolin; L) for 0.5 h and then stimulated with 1 mM IS (IS) for 6 h. (a) Relative mRNA expression of *AhRR* and *Cyp1A1* was measured using real-time RT-PCR; r*18S RNA* was used as a reference gene. Values are mean ± SE (*n* = 3–4). Different letters indicate a significant difference between groups. (b) Cytosol and nuclear levels of AhR (arrows) were analyzed using western blotting. Representative blots are shown.

To evaluate whether treatment with the tested flavones suppresses IS-induced AhR activation, the expression levels of representative AhR-responsive genes, cytochrome P450 1A1 (*CYP1A1*), and aryl hydrocarbon receptor repressor (*AhRR*), were examined. Six-hour incubation of HUVECs with 1 mM IS markedly increased mRNA expression of these genes. When the cells were pretreated with each of the three flavones, apigenin, chrysin, or luteolin (these flavones were removed before adding IS), IS-induced increases in the expression of these genes were significantly suppressed (Fig. 2a). Consistent with this, nuclear levels of AhR were increased by IS treatment, indicating activation of AhR, while pre-incubation with flavones suppressed the IS-induced intranuclear translocation of AhR (Fig. 2b).

### Apigenin, chrysin, and luteolin suppressed IS-induced increase in the expression of genes involved in endothelial dysfunction

**Fig. 3:**
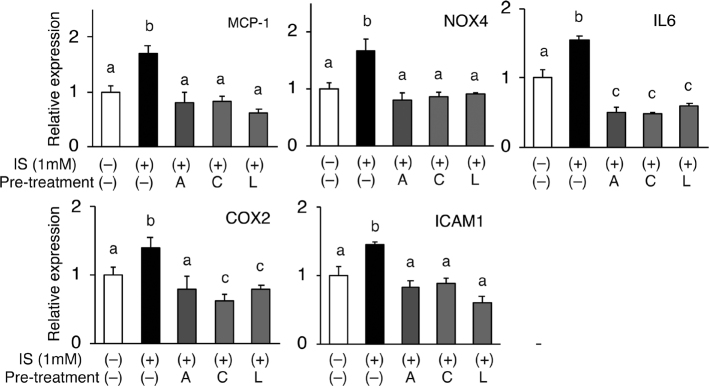
Effects of IS and each flavone on the expression of genes involved in inflammation and cell adhesion. HUVECs were pretreated with the indicated flavones (apigenin; A, chrysin; C, luteolin; L) for 0.5 h and then stimulated with 1mM IS (I) for 6 h. mRNA expression of *NOX-4, MCP-1, IL6, COX-2,* and *ICAM-1* was measured using real-time RT-PCR; r*18S RNA* was used as a reference gene. Values are the mean ± SE (*n* = 3–4). Different letters indicate a significant difference between groups.

Several previous studies reported that IS treatment alters the expression of genes involved in endothelial dysfunction (11–13, 16–18). Consistent with these reports, the treatment of HUVECs with IS significantly increased the expression of *NOX-4, MCP-1, IL6, COX-2,* and *ICAM-1* (Fig. 3). IS-induced increases in the expression of these genes were significantly suppressed by pretreating the cells with each flavone (Fig. 3).

### Apigenin, chrysin, and luteolin suppressed ROS production and STAT3 signaling in IS-stimulated HUVECs

**Fig. 4:**
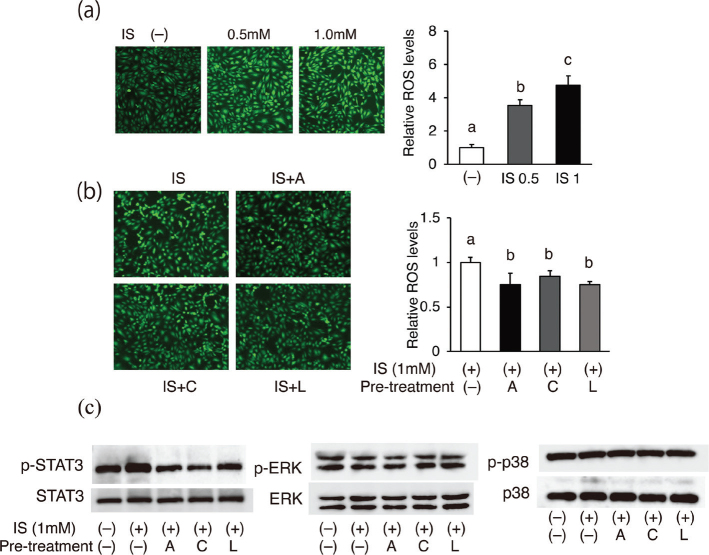
Effects of IS and each flavone on ROS production. (a) Cells were treated with 0, 0.5, or 1 mM of IS for 6 h. (b) The cells were pretreated with the indicated flavones (apigenin; A, chrysin; C, luteolin; L) for 0.5 h and then stimulated with 1 mM IS (I) for 6 h. Representative images of cells treated with CM-H2DCFDA are shown. Relative ROS levels were quantified as fluorescent intensities. Values are mean ± SE (*n* = 3–4). Different letters indicate a significant difference between groups. (c) The protein phosphorylation level of each signaling molecule was analyzed using western blotting. Representative blots are shown.

IS-induced increases in ROS levels were reported to play a central role in inflammatory gene expression by activating NF-κB via the ERK/p38MAPK pathway (11, 12, 38). Therefore, we examined whether apigenin, chrysin, and luteolin inhibit IS-induced ROS production in HUVECs. Intracellular ROS production was highly increased by IS treatment (0.5 or 1 mM), showing obvious green fluorescence (Fig. 4a). Pretreatment of cells with 10 μM of each flavone resulted in significant suppression of this increase in ROS production after treatment with 1 mM IS (Fig. 4a). However, neither IS nor the flavones affected ERK and p38 activity, as assessed by phosphorylation levels (Fig. 4b). In contrast, IS treatment consistently increased the protein levels of phosphorylated STAT3, which was reported to be involved in IS-induced pro-inflammatory effects through AhR (39, 40), and all three flavones inhibited IS-induced STAT3 phosphorylation (Fig. 4b).

### Chrysin ameliorated blood parameters and pathological damages in IS-induced renal failure in mice

**Fig. 5:**
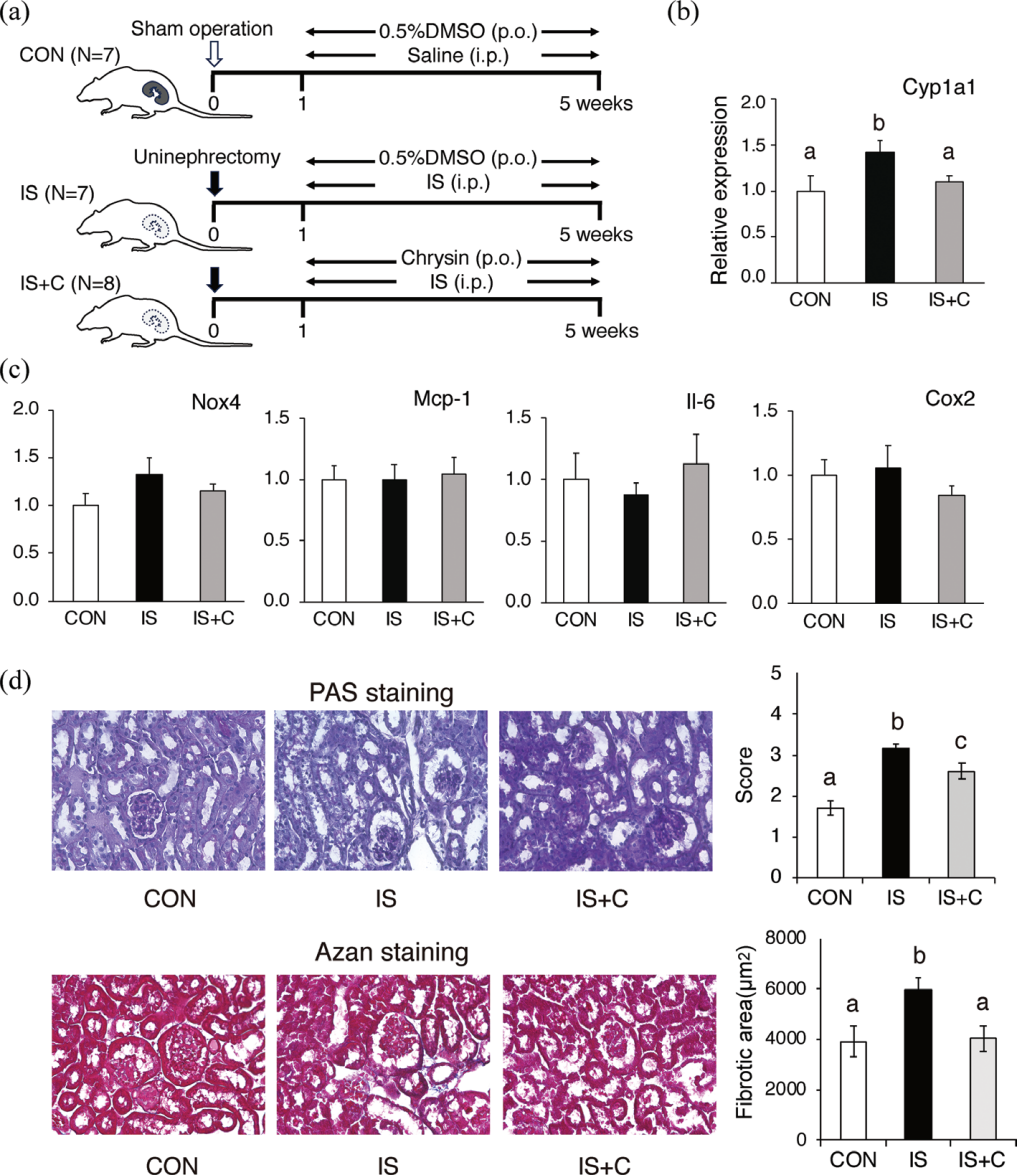
Effect of oral chrysin administration on IS-induced renal injury in mice. (a) Experimental scheme of the study. p.o.; oral administration (per os), i.p.; intraperitoneal administration. (b, c) Kidney tissue was harvested after 4 weeks of experiment and mRNA levels of (b) *Cyp1A1,* and (c) *Nox-4, Mcp-1, Il6,* and *Cox-2* were measured using real-time RT-PCR. *Gapdh* was used as a reference gene. (d) Representative light micrographs of kidney sections stained with PAS stain (top) or Azan stain (bottom) are shown. Morphological damage was scored based on the PAS-stained images, and fibrotic areas were quantified based on the AZAN-stained images. Values are mean ± SE (*n* = 7–8). Different letters indicate a significant difference between groups.

Microvascular damage caused by uremic toxins such as IS is considered a major cause of renal function deterioration. Therefore, we investigated the protective effects of chrysin against IS-induced renal injury. The experimental protocol for the 5-week experimental period is shown in Fig. 5a. Prolonged intraperitoneal injection of IS significantly increased serum IS and BUN levels (Table 1) and renal gene expression of *Cyp1a1* (Fig. 5b), a representative AhR target gene, in mice in the IS group receiving unilateral nephrectomy. Oral chrysin administration inhibited the increases in BUN levels and renal gene expression of *Cyp1a1*, although the serum levels of IS were not suppressed (Table 1, Fig. 5b). Expression of *Ahrr*, another representative AhR target gene, was not detected in kidney tissues (data not shown). We also examined the expression of inflammatory genes (*Nox4*, *Mcp-1*, *Il-6,* and *Cox2*) upregulated by IS *in vitro*; however, no significant differences were observed among the three groups (Fig. 5c).

**Table 1:** Body weight, kidney weight, and blood parameters

	CON group (*n* = 7)	IS group (*n* = 7)	IS+C group (*n* = 8)
Body weight (g)	28.3 ± 0.88	27.6 ± 0.33	27.0 ± 0.48
Kidney weight (g)	0.217 ± 0.018	0.206 ± 0.006	0.216 ± 0.011
Serum IS (mg/dl)	0.096 ± 0.024	0.310 ± 0.070*	0.262 ± 0.051*
BUN (mg/dl)	24.90 ± 2.64	42.07 ± 1.22*	30.90 ± 1.85*^§^

*P*-values below 0.05 are significant.

* *P* < 0.05 compared with CON group;

§ *P* < 0.05 compared with IS group.

Histopathological examination of kidney tissues from the IS group showed morphological damage, including brush border loss, tubule dilatation, tubule degeneration, and dilation of the Bowman’s capsule (Fig. 5d). In contrast, such injury features were attenuated in the kidneys of mice in the IS + C group compared with those in the IS group. Interstitial fibrosis was also observed in the renal tissues of mice in the IS group, whereas fibrotic areas were significantly reduced in the IS + C group (Fig. 5d).

## Discussion

Increasing evidence suggests that uremic toxins metabolized from dietary tryptophan such as IS and IAA are involved in CKD development. Many studies have demonstrated that IS induces deleterious effects such as inflammation, ROS production, and cell death in endothelial cells (see review (41)), suggesting that the activation of AhR is mechanistically involved (13, 25, 26, 29). AhR is a nuclear receptor that was originally reported to be activated by halogenated aromatic hydrocarbons such as TCDD and mediates many of the toxic effects of these chemicals (20, 23). Recent evidence shows that tryptophan-derived uremic toxins activate this receptor in a similar manner. Activated AhR binds to its responsive element in the promoter region of the target genes, *CYP1A1* and *AHRR*, an AhR repressor that competes for AhR binding to DNA, leading to a negative feedback loop for AhR (26, 42, 43). Furthermore, several natural food polyphenols have been reported to inhibit the TCDD-mediated activation of AhR (35, 36); however, few studies have examined whether these polyphenols regulate AhR activation induced by uremic toxins. The present results suggest that antagonistic polyphenols for TCDD-induced activation of AhR also exert inhibitory effects on IS-mediated activation in endothelial cells, thereby inhibiting the mRNA expression of AhR-targeted genes.

The mechanism by which AhR is activated by IS and causes inflammation is not fully understood. A previous study reported that IS increased E-selectin expression in an AhR-dependent manner in HUVECs; however, AhR did not directly bind to the E-selectin gene promoter (29). Another study demonstrated that IAA, another uremic toxin, upregulated the gene expression of tissue factor (TF), an activator of blood coagulation; this increase was attenuated by AhR knockout; however, AhR did not bind to the TF gene promoter (31). These results suggest that AhR activated by IS leads to the activation of specific intracellular signaling molecules thus increasing the expression of inflammatory genes rather than directly enhancing the promoter activity of these genes as a transcription factor. Several previous reports suggested that high ROS production levels mediated by IS result in the upregulation of inflammatory gene expression through ROS-dependent ERK/MAPK activation (11, 38). Indeed, ROS production was significantly increased in HUVECs treated with IS, and these increases were attenuated by all flavones tested in this study. However, neither IS nor the three flavones affected ERK and p38 MAPK activation. More recent studies have shown that STAT3 is involved in the signaling pathway through which IS-induced AhR activation exerts its biological action *in vitro* (39, 40, 44). Consistent with these reports, our results suggest that three flavones suppress IS-induced inflammatory responses by inhibiting the action of AhR, which causes STAT3 activation.

We also investigated the effects of IS on renal function *in vivo*. Several procedures have been reported to generate animal CKD models; among them, the administration of renal-toxic drugs or 5/6 nephrectomy has widely been used (45). However, in mice with toxic compound-induced CKD, the direct effect of the drug on the initiation of inflammation cannot be eliminated. Alternatively, mice that underwent 5/6 nephrectomy presented high mortality after modeling owing to hemorrhage and infection during or after surgery. Therefore, we attempted to generate a mouse model of CKD with high serum IS levels by referring to a study by Sun et al. (46). Daily intraperitoneal injection of IS into uninephrectomized mice for 4 weeks resulted in high serum concentrations of IS and BUN, which are associated with histopathological damage and renal fibrosis. In this model, the elevation of serum creatinine was not apparent and is consistent with previous results obtained using uninephrectomized mice (47). We used this model to assess the direct effect of IS on the kidneys.

In the animal studies, we selected chrysin, one of the three flavones tested *in vitro,* and investigated the effects of chrysin administration on CKD using the aforementioned animal model. We selected chrysin for various reasons. First, flavones have low solubility in aqueous buffer and low bioavailability *in vivo*; thus, for administration, highly concentrated flavone solutions with organic solvents have to be prepared. Chrysin is better soluble in organic solvents, including DMSO, than other flavones. Thus, the administered amount of DMSO, which can be toxic to mice, could be lower in our chrysin treatment. Second, chrysin has ‘lower’ antioxidant properties than other flavones (48). The purpose of this study was to investigate the anti-inflammatory effects of flavones via inhibition of AhR activation rather than via the direct antioxidant effects of flavones themselves, which were minimized by using chrysin.

Oral administration of chrysin significantly decreased BUN levels and ameliorated pathological damage to kidney tissues, despite comparable levels of serum IS in chrysin-treated and untreated mice. Combined with the results revealing that the increased expression of *Cyp1a1* observed in the IS group was suppressed in the IS + C group, chrysin might prevent IS-induced renal injury by suppressing AhR activation due to increases in serum IS levels. Chrysin is a metabolite found in various medicinal plants and widely used in traditional medicine (49). Many studies have reported protective effects of chrysin against kidney failure in different rodent models (50–52). However, these studies have used CKD models developed by adding toxic substances to animals and have assumed the radical scavenging effect of hydroxyl groups at positions 5 and 7 in this flavone as the underlying mechanism. The present results suggest a novel mechanism by which chrysin ameliorates CKD pathogenesis by counteracting the effects of IS through its suppressive effects on AhR. In contrast, in animal experiments, the expression levels of cytokine genes regulated by IS and flavones *in vitro* did not differ among the three groups. This may have resulted from the heterogeneity of kidney tissue, which consists of multiple cell components, including tubular epithelial, mesenchymal (fibroblasts and pericytes), endothelial, and inflammatory cells. Nguyen et al. demonstrated increased expression of CYP1A1 and NOX4 specifically in the aortic ring endothelium of IS-exposed rats using immunostaining (53). Therefore, the levels of region-specific gene expression, especially in the vascular region of renal tissue, need to be investigated.

This study has several limitations. First, we did not directly evaluate whether the three flavones inhibit IS-induced increases in *AhRR* and *Cyp1A1* by binding to AhR in a competitive manner with IS. However, this mechanism appears reasonable since these flavones were reported to competitively inhibit the activation of AhR by dioxin, a major ligand, at the concentrations used in this study (36). This requires further investigation. Second, the sample size in the cellular experiments was somewhat limited (*n* =3 to 4). Although we repeated the experiments at least twice independently and confirmed that the same results were obtained, an increase in sample size will be required in future experiments. Third, we cannot exclude the possibility that chrysin ameliorated renal dysfunction via its antioxidant properties rather than via an antagonistic effect on AhR activation. Additionally, we did not examine the *in-vivo* efficacy of the other two flavones apigenin or luteolin in this study. Further studies are required to evaluate the effect of these flavones on IS-induced renal dysfunction.

The present study revealed that three flavones, apigenin, chrysin, and luteolin, inhibited IS-induced pro-inflammatory changes, which was characterized by an increase in the gene expression of inflammatory cytokines and chemokines (*NOX-4, MCP-1*, *IL-6*, and *COX-2*), an adhesion molecule (*ICAM-1*), and ROS production in endothelial cells, by suppressing AhR-induced activation of STAT3. In addition, oral administration of chrysin attenuated the deterioration of renal failure induced by IS in mice, probably via its suppressive effect on IS-mediated AhR activation. Patients with CKD are at high risk of cardiovascular complications; thus, there is increasing interest in the deteriorating effects of uremic toxins and AhR on vascular inflammation. The vascular endothelium is both a target and driver, not only for kidney impairment but also for cardiovascular complications. Therefore, the potential effects of the flavones tested in this study could be a therapeutic strategy for targeting vascular inflammation in patients with CKD.

## Supplementary Material


