# Supplementary material for: Natural antagonistic flavones for AhR inhibit indoxyl sulfate-induced inflammatory gene expression in vitro and renal pathological damages in vivo
Source: Food Nutr Res. 2024 Jul 31;68:10.29219/fnr.v68.10032. doi: 10.29219/fnr.v68.10032 (PMC11305152; doi:10.29219/fnr.v68.10032)
Supplement: Supplementary file 1 [file FNR-68-10032-s1.docx]

|  | Forward | Reverse |
| --- | --- | --- |
| (Human) |  |  |
| *r18S* | ACTCAACACGGGAAACCTCAC | CAGACAAATCGCTCCACCAA |
| *CYP1A1* | TGCCAAGAGTGAAGGGAAGAG | GAAGGGCAGAGGAATGTGATG |
| *AhRR* | GCAAAACCCAGAGCAGACAC | TGTTCCCTGAGCACCAAAAC |
| *NOX4* | GCTGTATAACCAAGGGCCAGA | TCGGAGGTAAGCCAAGAGTG |
| *MCP-1* | ATCACCAGCAGCAAGTGTCC | CAAGTCTTCGGAGTTTGGGTTT |
| *IL-6* | ACCTTCCAAAGATGGCTGAAAA | TGGCTTGTTCCTCACTACTCTCA |
| *COX2* | CCAGCACTTCACGCATCAGT | ACGCTGTCTAGCCAGAGTTTCAC |
| *ICAM-1* | GGGCAGTCAACAGCTAAAACCTT | CACCTGGCAGCGTAGGGTAA |
| (Mouse) |  |  |
| *Gapdh* | TGACGTGCCGCCTGGAGAAA | AGTGTAGCCCAAGATGCCCTTCAG |
| *Cyp1a1* | GGTTAACCATGACCGGGAACT | TGCCCAAACCAAAGAGAGTGA |
| *Nox4* | TGCCTGCTCATTTGGCTGT | CCGGCACATAGGTAAAAGGATG |
| *Mcp-1* | ATGCTTCTGGGCCTGCTGT | GGATCATCTTGCTGGTGAATGAG |
| *Il-6* | ACAACCACGGCCTTCCCTACTT | CACGATTTCCCAGAGAACATGTG |
| *Cox2* | CAAGACAGATCATAAGCGAGGA | GGCGCAGTTTATGTTGTCTGT |

Table S1 Primer sequences
